# Profiling the low-beta characteristics of the subthalamic nucleus in early- and late-onset Parkinson’s disease

**DOI:** 10.3389/fnagi.2023.1114466

**Published:** 2023-02-16

**Authors:** Delong Wu, Baotian Zhao, Hutao Xie, Yichen Xu, Zixiao Yin, Yutong Bai, Houyou Fan, Quan Zhang, Defeng Liu, Tianqi Hu, Yin Jiang, Qi An, Xin Zhang, Anchao Yang, Jianguo Zhang

**Affiliations:** ^1^Department of Neurosurgery, Beijing Tiantan Hospital, Capital Medical University, Beijing, China; ^2^Beijing Key Laboratory of Neurostimulation, Beijing, China; ^3^Department of Functional Neurosurgery, Beijing Neurosurgical Institute, Capital Medical University, Beijing, China

**Keywords:** Parkinson’s disease, aging, subthalamic nucleus, microelectrode recording, local field potential

## Abstract

**Objectives:**

Low-beta oscillation (13–20 Hz) has rarely been studied in patients with early-onset Parkinson’s disease (EOPD, age of onset ≤50 years). We aimed to explore the characteristics of low-beta oscillation in the subthalamic nucleus (STN) of patients with EOPD and investigate the differences between EOPD and late-onset Parkinson’s disease (LOPD).

**Methods:**

We enrolled 31 EOPD and 31 LOPD patients, who were matched using propensity score matching. Patients underwent bilateral STN deep brain stimulation (DBS). Local field potentials were recorded using intraoperative microelectrode recording. We analyzed the low-beta band parameters, including aperiodic/periodic components, beta burst, and phase-amplitude coupling. We compared low-beta band activity between EOPD and LOPD. Correlation analyses were performed between the low-beta parameters and clinical assessment results for each group.

**Results:**

We found that the EOPD group had lower aperiodic parameters, including offset (*p* = 0.010) and exponent (*p* = 0.047). Low-beta burst analysis showed that EOPD patients had significantly higher average burst amplitude (*p* = 0.016) and longer average burst duration (*p* = 0.011). Furthermore, EOPD had higher proportion of long burst (500–650 ms, *p* = 0.008), while LOPD had higher proportion of short burst (200–350 ms, *p* = 0.007). There was a significant difference in phase-amplitude coupling values between low-beta phase and fast high frequency oscillation (300–460 Hz) amplitude (*p* = 0.019).

**Conclusion:**

We found that low-beta activity in the STN of patients with EOPD had characteristics that varied when compared with LOPD, and provided electrophysiological evidence for different pathological mechanisms between the two types of PD. These differences need to be considered when applying adaptive DBS on patients of different ages.

## 1. Introduction

Parkinson’s disease (PD) is a movement disorder characterized by bradykinesia and at least one of resting tremor and rigidity, as well as a variety of non-motor symptoms (Postuma et al., 2015). PD can be classified into two subtypes according to age of onset: early-onset PD [EOPD, age of onset ≤50 years (Butterfield et al., 1993; Schrag and Schott, 2006; Mehanna and Jankovic, 2019; Niemann and Jankovic, 2019), although some studies define the upper age limit as 40 years (Quinn et al., 1987; Schrag et al., 2000)] and late-onset PD (LOPD). Patients with EOPD typically have poorer social adjustment, higher rates of depression, and inferior quality of life compared to LOPD patients, as it affects those who are in the prime of their productivity (Mehanna and Jankovic, 2019). EOPD patients may experience more severe physical, financial, and psychological problems by the time they reach the age of LOPD patients, because of their longer disease duration. Therefore, maintaining daily social and occupational functioning is the treatment focus in EOPD, while postponing or ameliorating motor complications of treatment, offering psychological support, and, if possible, preventing psychiatric problems such as anxiety and depression (Schrag and Schott, 2006; Niemann and Jankovic, 2019).

Although current medication can provide good symptomatic remission, patients will still develop motor complications and fluctuations as the disease progresses. Deep brain stimulation (DBS) is a well-established treatment for PD, and provides a unique opportunity to gain insights into local field potentials (LFPs), which are recorded from the neuron population surrounding the target area by the depth electrodes (Okun, 2012; Fox et al., 2018). Aiming to save energy and reduce side effects, adaptive DBS (aDBS) automatically trims stimulation depending on neurophysiological feedback, in which sensitive and specific electrophysiological biomarkers play a vital role (Rosin et al., 2011; Swann et al., 2018).

In recent years, aperiodic components of spectra have been noticed in electrophysiological signal analyze, which was considered as 1/*f*-like instructed noise before. In oscillatory analysis, reliance on *a priori* frequency bands may lead to the inclusion of aperiodic activity from outside the real physiological oscillation range (Donoghue et al., 2020). Studies have proved that there is correlation between age and this 1/*f*-like component (Voytek et al., 2015). Many researchers have found that beta activity could potentially be a feedback signal for aDBS, owing to its correlation with parkinsonian symptom severity and because it can be regulated by medical treatment and DBS. Evidence has shown that low-beta activity is more dominant within the subthalamic nucleus (STN) and is regarded as a pathological oscillation (Tsiokos et al., 2017). In addition, low beta has been proved to be more sensitive to dopaminergic or STN DBS (Litvak et al., 2011; Neumann et al., 2016a). Furthermore, beta burst has been proved to have a closer relationship with motor impairment and etiology of PD (Tinkhauser et al., 2017a; Torrecillos et al., 2018; Lofredi et al., 2019a,b). Moreover, a recent study with a large cohort of 106 PD patients shows similar results that both band power and burst duration of frequency-specific low beta (13–20 Hz) have significant correlations with motor symptom severity, and the dopamine-related reduction of band power and burst duration are paralleled by dopamine-related symptom alleviation (Lofredi et al., 2023). Phase-amplitude coupling (PAC) may be interpreted considerably differently between STN PAC and cortical PAC, as cortical PAC involves the amplitude of broadband activity (50–200 Hz), not an oscillatory rhythm, whereas STN PAC involves the amplitude of high frequency oscillation (HFO, 200–500 Hz). Beta/HFO PAC in the basal ganglia has been found to be correlated to motor impairment severity (Lopez-Azcarate et al., 2010). Another study has similar findings, and furthermore, this effect was more pronounced within the low-beta range, while coherence between subthalamic nucleus and motor cortex was dominant in the high-beta range (van Wijk et al., 2016). However, most previous studies on PD electrophysiology have obtained signals from elderly patients. It is noted that most EOPD cases result from Lewy body like LOPD, or less commonly, gene mutation (Schrag and Schott, 2006). Nevertheless, EOPD has the characteristics such as increased genetic predisposition, slower progression, and increased risk of levodopa-related complications, when compared to LOPD (Niemann and Jankovic, 2019). So we hypothesized that electrophysiological features of low beta in the STN of patients with EOPD would be different from those of patients with LOPD. This study aimed to explore the low-beta oscillation characteristics of the STN in patients with EOPD, to promote the development of aDBS in PD. In particular, we separated the aperiodic and periodic components, and addressed the differences in low-beta burst and beta/HFO PAC in the STN between patients with EOPD and those with LOPD.

## 2. Materials and methods

### 2.1. Patient inclusion

A total of 202 consecutive patients diagnosed with PD who underwent STN-DBS surgery between December 2019 and January 2021 at Tiantan Hospital were sampled. In our center, patients who showed dyskinesia or other L-dopa related complications were recommended to STN DBS, while patients with cognitive decline or mental disorder were prone to globus pallidus internus (GPi) DBS (Rughani et al., 2018). The inclusion criteria were (1) diagnosis of idiopathic PD, according to the United Kingdom Parkinson’s Disease Society Brain Bank Clinical Diagnostic Criteria, (2) bilateral STN-DBS surgery was performed, and (3) preoperative clinical assessments were completed, including minimum demographic information; age of disease onset; disease duration; levodopa equivalent daily dose (LEDD); Movement Disorders Society Unified Parkinson’s Disease Rating Scale (MDS-UPDRS) including medication (Med) ON and OFF; Hamilton Anxiety Scale (HAMA), Hamilton Depression Scale (HAMD); Beijing version of Mini-Mental State Examination (MMSE; Li et al., 2016); and Chinese version of Montreal Cognitive Assessment (MoCA; Huang et al., 2018; permission was obtained at https://www.mocatest.org/). Excluded were (1) patients who had received any other intracranial surgery previously and (2) patients who had severe surgery-related complications such as cerebral hemorrhage and hemiplegia. Patients were matched in terms of clinical baseline by propensity score matching (PSM; details in Statistical Analysis section). Patients gave informed written consent, and the study was approved by the institutional review board of Beijing Tiantan Hospital.

### 2.2. Surgical procedures and signal recording

Patients were operated by the same team and a standard surgical procedure was conducted as previously reported (Fan et al., 2020; Xie et al., 2022). Briefly, the STN target coordinates and trajectory were determined using a surgical planning system (Surgiplan, Elekta Instrument AB, Stockholm, Sweden) with which we combined the CT scan with a stereotactic frame and 3-D high resolution magnetic resonance (MR) images, preoperatively. The STN target coordinates for the lower contact were 2–3 mm posterior to the mid commissural point (MCP), 12–14 mm lateral to the anterior commissure–posterior commissure (AC-PC), and 4–6 mm below the inter-commissural line. We performed intraoperative microelectrode recording (MER) using a tungsten microelectrode (10–20 μm at the tip with impedance 0.4–1 MΩ, Alpha Omega Engineering, Nazareth, Israel) and the Neuro-Omega system (Alpha Omega Engineering, Nazareth, Israel), sampled at 44 kHz. MER was started at 10 mm above the target and advanced in small discrete steps (stepwise ranging from 0.1 to 0.5 mm), controlled by the neurophysiologist to achieve optimal recording and identification of the borders of the STN. The STN was identified visually as characterized by a prominent increase in background activity with typical multiple neuronal discharges of high frequency. During MER, patients were awake and not under sedation. Data were obtained in the resting state and at least 12 h since last dopaminergic medication. Macro-stimulation was then performed to confirm the target position, testing the effect on motor symptom control and observing for any side effects. The programmable pulse generator (IPG) was then implanted in the subclavicular area under general anesthesia. Postoperative CT was performed to exclude intracranial hemorrhage and the exact locations of the DBS leads verified by merging with preoperative MR images.

### 2.3. Electrophysiological data processing

Data preprocessing was performed in MATLAB (Version 2020b, MathWorks, United States) with customized scripts and the Fieldtrip toolbox (Oostenveld et al., 2011).^1^ All electrophysiological data were manually visualized offline and recordings with obvious artifacts were discarded. Artifact-free LFP data from all the recording depths inside STN were accumulated for further analysis. The electrophysiology data were downsampled to 2000 Hz and a notch filter was applied to the data to remove the 50 Hz line noise and harmonics. Data were z-score normalized for subsequent process and analysis.

#### 2.3.1. Aperiodic and periodic component

**Figure 1:**
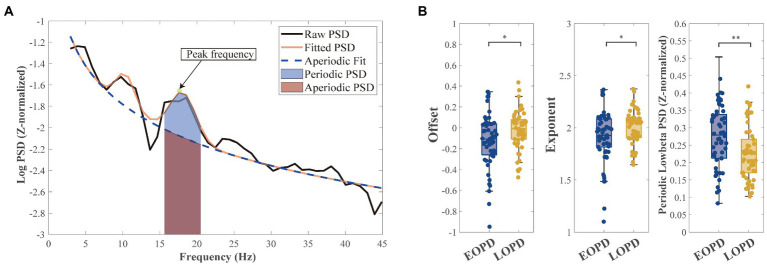
Schematic diagram of parameterization of power spectra. **(A)** An example of parameterizing the power spectra: the aperiodic component (blue dashed line) and the fitted spectra (orange full line). Mixed power is equal to the sum of periodic power (light blue shadow) and aperiodic power (light purple shadow). Peak frequency (green point and arrowed) in low-beta band with highest power was selected for subsequent burst analysis. **(B Left)** Early-onset Parkinson disease (EOPD) patients demonstrated lower offset than late-onset Parkinson disease (LOPD) patients (EOPD: −0.14 ± 0.28, LOPD: −0.02 ± 0.19, *p* = 0.010), meaning EOPD patients had a lower spectrum curve. **(B Middle)** EOPD patients demonstrated lower exponent than LOPD patients (EOPD: 1.92 ± 0.25, LOPD: 2.00 ± 0.16, *p* = 0.047), meaning LOPD patients had a steeper spectrum curve. **(B Right)** After separating the aperiodic components from the periodic components, we observed periodic low-beta power that was significantly higher in EOPD patients than LOPD patients (EOPD: 0.26 ± 0.09, LOPD: 0.22 ± 0.08, *p* = 0.002).

The data were filtered between 2 and 500 Hz using a zero-phase third-order Butterworth bandpass filter. The power spectral density (PSD) was calculated in each trace using Welch’s method with a 1 s Hanning window (50% overlap). We separated the aperiodic and periodic component of the LFP signal using the FOOOF algorithm (Donoghue et al., 2020). We set the FOOOF parameters as follows: peak width limits: 2–12; maximum number of peaks: infinite; minimum peak height: 0; peak threshold: 2; and aperiodic model: fixed. The Welch’s PSD was fitted into the FOOOF model and parameterized across the frequency range of 2–45 Hz, which separated the aperiodic components (offset and exponent) from the periodic components, which included peak frequency (arrow in Figure 1A) for further analysis of beta burst. After the parameterization of the power spectra, the fitted spectra were used to subtract the aperiodic power (area under the aperiodic fit curve) to obtain periodic PSD (blue shadow in Figure 1B).

#### 2.3.2. Low-beta burst

**Figure 2:**
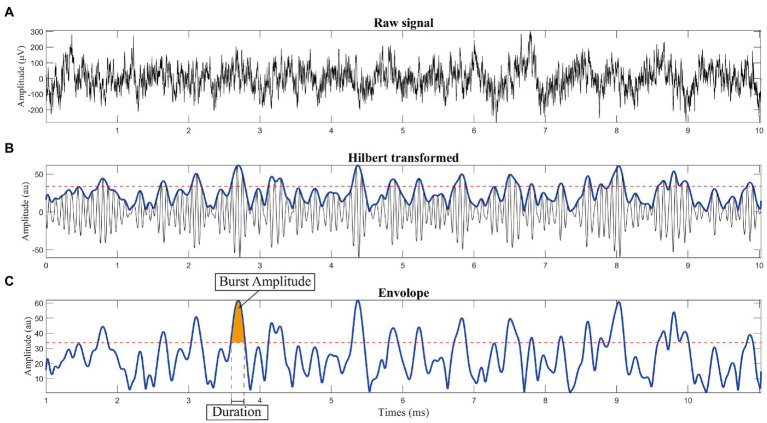
Schematic diagram of burst analysis. **(A)** Raw signal recorded from the subthalamic nucleus using microelectrode recoding. **(B)** Calculating the envelope: we used the Hilbert transform with a 6 Hz bandwidth centered around the selected peak frequency (peak frequency is shown in Figure 1A). **(C)** Threshold was defined in terms of the 75th percentile of the Hilbert envelope amplitude, as shown by the red dashed line. Burst was identified as wavelet amplitude exceeding the applied amplitude threshold. Burst duration was defined by the time points at which the selected time evolution of the wavelet amplitude exceeded a given amplitude threshold. To reduce the contribution of other noise, bursts with durations shorter than 100 ms were excluded before further analysis.

**Figure 3:**
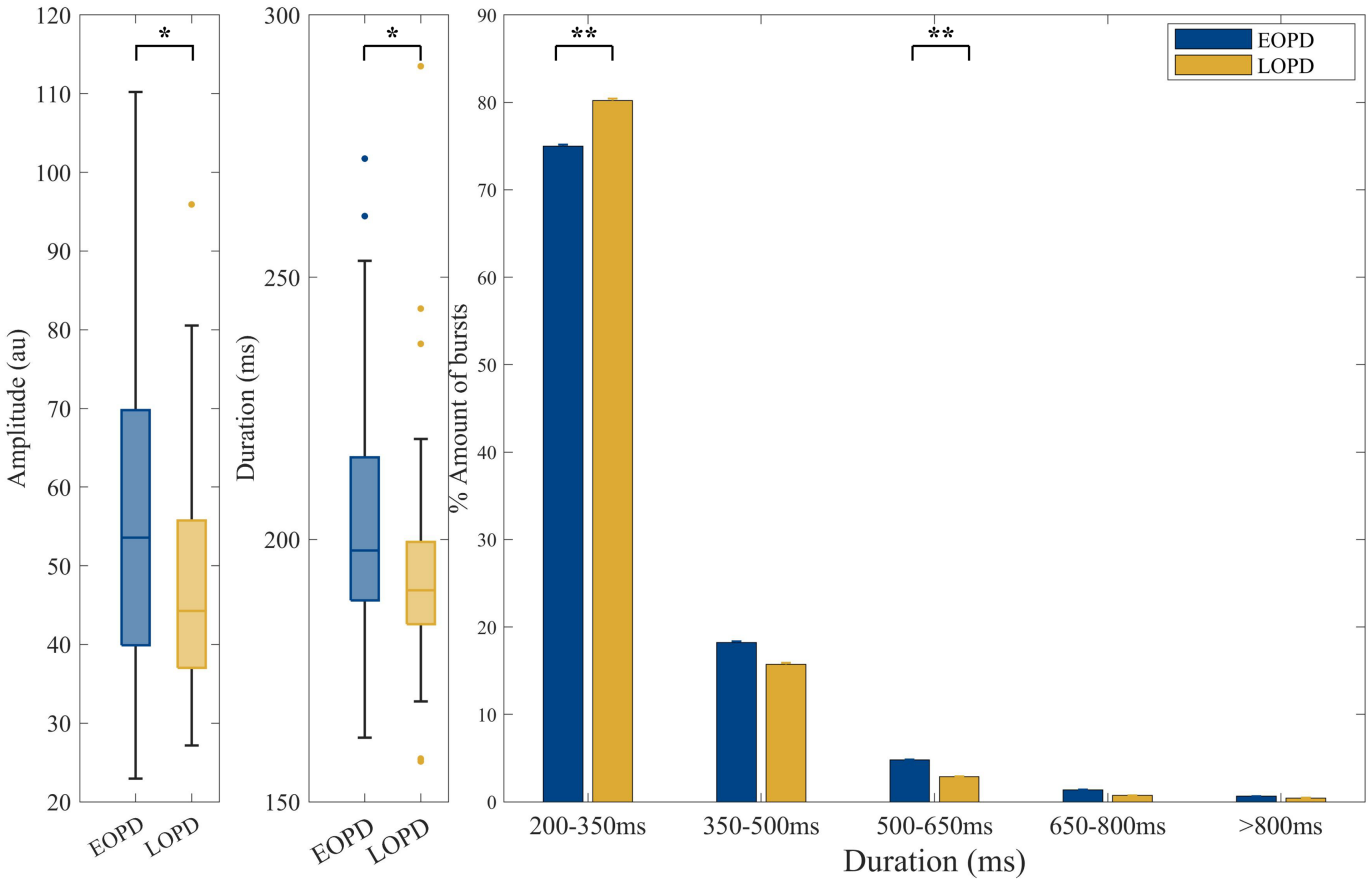
Comparison of low-beta burst between EOPD and LOPD. **(A)** The average low-beta burst power was significantly higher in EOPD (EOPD: 56.70 ± 20.10 au. LOPD: 47.27 ± 14.73 au, *p* = 0.016). **(B)** The average low-beta burst duration of EOPD was significantly longer than that of LOPD (EOPD: 203.74 ± 22.55 ms, LOPD: 193.79 ± 20.80 ms, *p* = 0.011). **(C)** Illustration of beta burst duration categorized into five time windows. The proportion of long burst (burst duration 500–650 ms) was significantly increased in EOPD when compared to LOPD (EOPD: 4.79% ± 3.86%, LOPD, 2.88% ± 2.87%, *p* = 0.008). In contrast, the proportion of short low-beta bursts (burst duration 200–350 ms) was significantly higher in LOPD when compared to EOPD (EOPD: 75.00% ± 10.52%, LOPD, 80.22% ± 10.79%, *p* = 0.007).

The criteria for burst determination generally followed a previous study (Tinkhauser et al., 2017b). The low-beta band power was represented as the averaged power across the corresponding frequency band (13–20 Hz). The beta peak frequency with the highest power of each recording trace, which was acquired from FOOOF peak identification, was selected. The envelope of the beta peak band filtered LFP was calculated using the Hilbert transform with a 6 Hz bandwidth centered around the selected peak frequency (beta peak band; Figure 2A). Threshold was defined in terms of the 75th percentiles of the Hilbert envelope amplitude (red dash line in Figures 2B,C). Beta burst was identified as wavelet amplitude exceeding the applied amplitude threshold. Bursts with durations shorter than 100 ms were excluded to limit the contribution of spontaneous fluctuations in amplitude due to noise (Deffains et al., 2018). The burst amplitude was defined as the area between the signal curve and threshold line (yellow shadow in Figure 2C). The distribution of burst durations was considered by categorizing them into five time windows of 150 ms, starting from 200 ms to >800 ms in duration (Lofredi et al., 2019a). Considering that the absolute number of bursts may have varied across traces, we calculated the percentage distribution of bursts in each time window, which served as a normalization step. The averaged amplitude and duration of all identified bursts were also calculated (Figure 3).

#### 2.3.3. Phase-amplitude coupling

PAC was calculated as we previously reported (Yin et al., 2022). We investigated 2–45 Hz as phase frequencies and 50–500 Hz range as amplitude frequencies. LFP data were bandpass filtered with a third-order Butterworth filter from 2 to 50 Hz with a 2 Hz bandwidth and 1 Hz shift, while the same LFP data were filtered from 50 to 500 Hz with a 4 Hz bandwidth and 2 Hz shift. Then, the instantaneous phase of the low frequency bandpass filtered signal and the instantaneous amplitude of the high frequency filtered signal were extracted using the Hilbert transform. We used the Kullback–Leibler distance, which measures the divergence between the probability distribution of high frequency amplitudes and uniform distribution, to calculate the modulation index (MI; Tort et al., 2010). The obtained MI was normalized by calculating the z-score of 200 surrogates generated by randomly swapping amplitude time blocks. Z-scored PAC computed for multiple frequencies of phase and amplitude were demonstrated as a comodulogram. The PAC calculations were conducted in Python 3 using the Tensorpac toolbox (Combrisson et al., 2020).^2^

### 2.4. Statistical analysis

We performed PSM to minimize the effects of potential confounding factors. Patients in this study were divided into two groups, EOPD group and LOPD group. Patients in the EOPD group were matched with a similar cohort of patients with LOPD (age of onset >50 years) in our dataset with a 1:1 ratio for disease duration, LEDD, MDS-UPDRS III scores (Med OFF), HAMA, HAMD, MMSE, and MoCA. PSM was performed using the nearest neighbor method within a caliper of 0.01 in SPSS (Version 27.0, IBM, United States).

Independent non-parametric tests (Wilcoxon rank-sum test) and Spearman’s correlation were used when the data were not normally distributed, which was tested by the Kolmogorov–Smirnov test; otherwise, independent Student’s *t*-tests and Person’s correlation were used. Correlation analysis was performed between age of onset and LFP characteristics in whole population. Correlation analysis was also performed between LFP characteristics and MDS-UPDRS III total and subdivided scores, including Med ON/OFF and improvement rate in whole population and within each groups, respectively. False Discovery Rate (FDR) correction was used for multiple comparison. A value of *p* <0.05 was considered significant. Statistical analyses were performed in MATLAB and R Studio (Version 1.4.17, PBC, United States).

$$\mathrm{improvement rate}=\;\left(\frac{Med\;\mathrm{OFF}\_Med\;\mathrm{ON}}{Med\;\mathrm{OFF}}\right)\;\times 100\%$$

## 3. Results

### 3.1. Patients

A total of 62 patients were enrolled including 31 with EOPD (11 female, 20 male) and 31 with LOPD (16 female, 15 male), matched through PSM. There were no significant differences between the two groups in preoperative clinical assessments such as disease duration (*p* = 0.963), LEDD (*p* = 0.086), MDS-UPDRS III scores (Med OFF, *p* = 0.832), HAMA (*p* = 0.216), HAMD (*p* = 0.061), MMSE (*p* = 0.140), and MoCA (*p* = 0.154). EOPD patients had a significantly higher improvement rate (*p* = 0.005) in MDS-UPDRS III scores, including total and some subdivided (bradykinesia and rigidity) scores, than the LOPD patients. The detailed patient demographics and clinical characteristics are summarized in Table 1. There was no significance difference in average signal duration between groups (EOPD: 229.39 ± 106.10 s, LOPD: 188.04 ± 125.29 s, *p* = 0.16).

**Table 1:** Demographic and clinical characteristics.

	EOPD	LOPD	*p* value
Age*	43.6 ± 6.3	60.0 ± 6.0	<0.001*
Gender(F/M)	11/20	16/15	
LEDD (mg/day)	936.26 ± 372.89	788.13 ± 289.25	0.086
Disease duration (years)	8.13 ± 2.60	8.16 ± 2.89	0.963
MDS-UPDRS	49.94 ± 16.36	49.03 ± 16.97	0.832
Part III (Med off)			
Axial	10.42 ± 4.65	10.26 ± 4.95	0.896
Bradykinesia	21.32 ± 7.46	20.71 ± 9.36	0.776
Rigidity	8.55 ± 3.01	7.81 ± 3.64	0.385
Tremor	9.65 ± 7.85	10.26 ± 6.64	0.741
MDS-UPDRS	*17.97 ± 8.86*	*25.00 ± 13.44*	0.018*
Part III (Med on)			
Axial	3.94 ± 3.04	5.39 ± 3.73	0.098
Bradykinesia	*7.90 ± 4.85*	*11.29 ± 7.26*	0.035*
Rigidity	3.03 ± 1.663	3.94 ± 2.41	0.091
Tremor	3.10 ± 2.95	4.39 ± 4.90	0.214
Improve rate (%)	*63.06 ± 17.77*	*51.07 ± 14.70*	0.005*
Axial	60.79 ± 25.01	49.28 ± 26.64	0.085
Bradykinesia	*62.37 ± 20.00*	*47.28 ± 17.17*	0.002*
Rigidity	*64.53 ± 18.35*	*50.19 ± 20.21*	0.005*
Tremor	70.73 ± 25.65	64.05 ± 28.22	0.356
HAMA	19.97 ± 8.94	16.90 ± 10.31	0.216
HAMD	20.03 ± 8.67	15.58 ± 9.64	0.061
MMSE	26.61 ± 5.36	24.77 ± 4.24	0.140
MoCA	22.35 ± 6.67	20.03 ± 5.99	0.154

Values are presented as mean ± standard deviation (SD). LEDD, Levodopa equivalent daily doses; MDS-UPDRS, Movement Disorders Society Unified Parkinson’s Disease Rating Scale; HAMA, Hamilton Anxiety scale; HAMD, Hamilton Depression scale; MMSE, Mini-mental State Examination; MoCA, Montreal Cognitive Assessment. MDS-UPDRS III off-medication evaluations were performed after the withdrawal of anti-parkinsonism medications for 8–12 h, and on-medication evaluations were performed when the patient had maximum clinical benefit following the patient’s regular dose of anti-parkinsonism medication.

* A significant difference.

### 3.2. Aperiodic components and periodic power spectral density

After parameterizing the LFP, we found significant differences in offset (EOPD: −0.14 ± 0.28, LOPD: −0.02 ± 0.19, *p* = 0.010) and exponent (EOPD: 1.92 ± 0.25, LOPD: 2.00 ± 0.16, *p* = 0.047) parameters between the two groups. Offset parameters represent broadband up/down shift of the whole spectrum, and our results showed that EOPD patients had a lower spectrum curve than LOPD patients. However, exponent represents the slope of the spectrum, which means in our study LOPD patients had a steeper spectrum curve than EOPD patients. By subtracting the aperiodic power with the fitted power, low-beta periodic PSD was found to be higher in EOPD patients (EOPD: 0.26 ± 0.09, LOPD: 0.22 ± 0.08, *p* = 0.002). The results are shown in Figure 1B.

### 3.3. Comparison of low-beta burst

The average amplitude of low-beta burst was significantly higher in EOPD (EOPD: 56.70 ± 20.10 au, LOPD: 47.27 ± 14.73 au, *p* = 0.016). The average low-beta burst duration of EOPD was significantly longer than that of LOPD (EOPD: 203.74 ± 22.55 ms, LOPD: 193.79 ± 20.80 ms, *p* = 0.011). Also, the ratio of long bursts (burst duration 500–650 ms) was significantly increased in EOPD when compared to LOPD (EOPD: 4.79% ± 3.86%, LOPD, 2.88% ± 2.87%, *p* = 0.008). In contrast, the proportion of short low-beta bursts (burst duration 200–350 ms) was significantly higher in LOPD when compared to EOPD (EOPD: 75.00% ± 10.52%, LOPD, 80.22% ± 10.79%, *p* = 0.007).

### 3.4. Beta/fHFO phase-amplitude coupling

**Figure 4:**
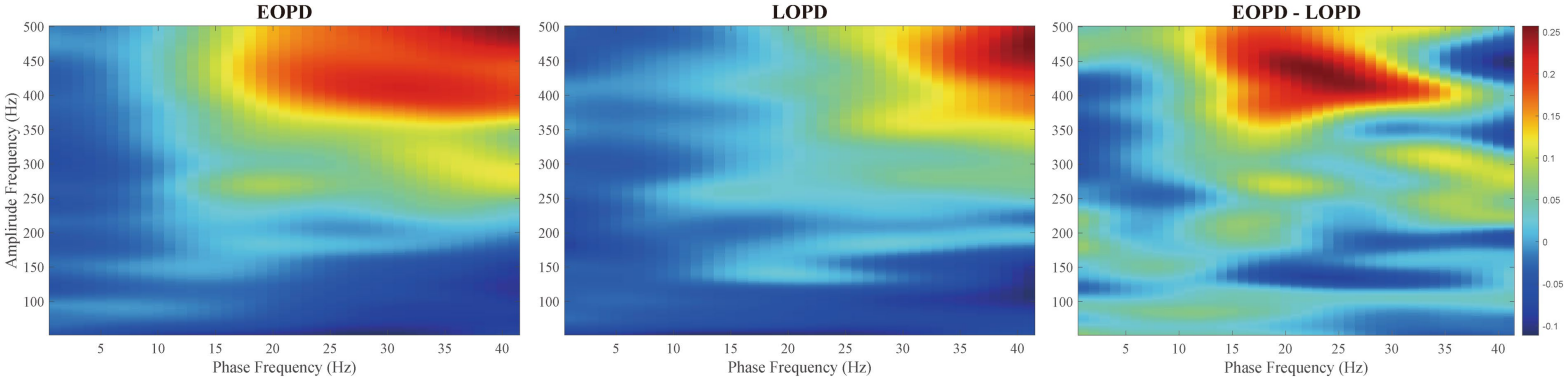
Comodulograms of phase-amplitude coupling. Modulation index (MI) was averaged across all EOPD **(Left)** and LOPD patients **(Middle)**, with phase frequency (range from 2 to 45 Hz) and amplitude frequency (range from 50 to 500 Hz) shown. **(Right)** Inter-group comparison showed a significant difference in MI of low beta phase (13–20 Hz) and fast HFO (fHFO, 300–460 Hz) amplitude coupling (*p* = 0.019).

The comodulograms of group-level PAC for EOPD and LOPD and their subtraction are shown in Figure 4. There was a significant difference between EOPD and LOPD groups in MI of low-beta phase (13–20 Hz) and fast HFO (fHFO, 300–460 Hz) amplitude coupling (*p* = 0.019), as shown in the right image of Figure 4.

### 3.5. Correlation analysis

**Figure 5:**
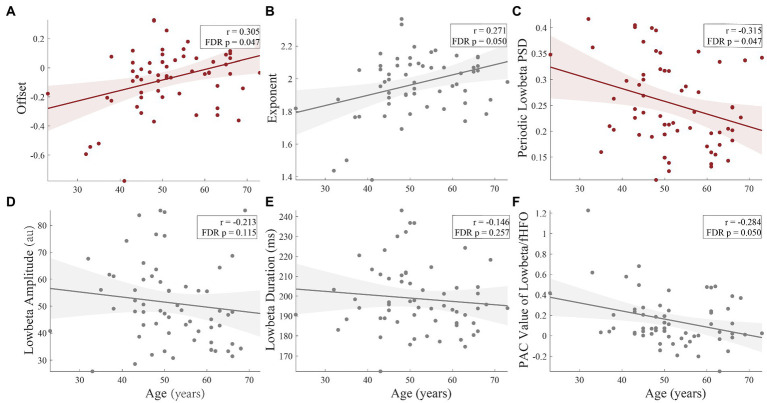
The correlations of age and the LFP parameters. There were significant correlations between age and offset (*r* = 0.305, FDR corrected *p* = 0.047), **(A)** and periodic low-beta PSD (*r* = −0.315, FDR corrected *p* = 0.047), **(C)** in whole population. Moreover, exponent (*r* = 0.271, FDR corrected *p* = 0.050), **(B)** and PAC value of low-beta/fHFO (*r* = −0.284, FDR corrected *p* = 0.050), **(E)** were marginal significantly correlated with age. The amplitude (FDR corrected *p* = 0.115) **(D)** and duration (FDR corrected *p* = 0.257) **(F)** had no significant correlations with age.

**Figure 6:**
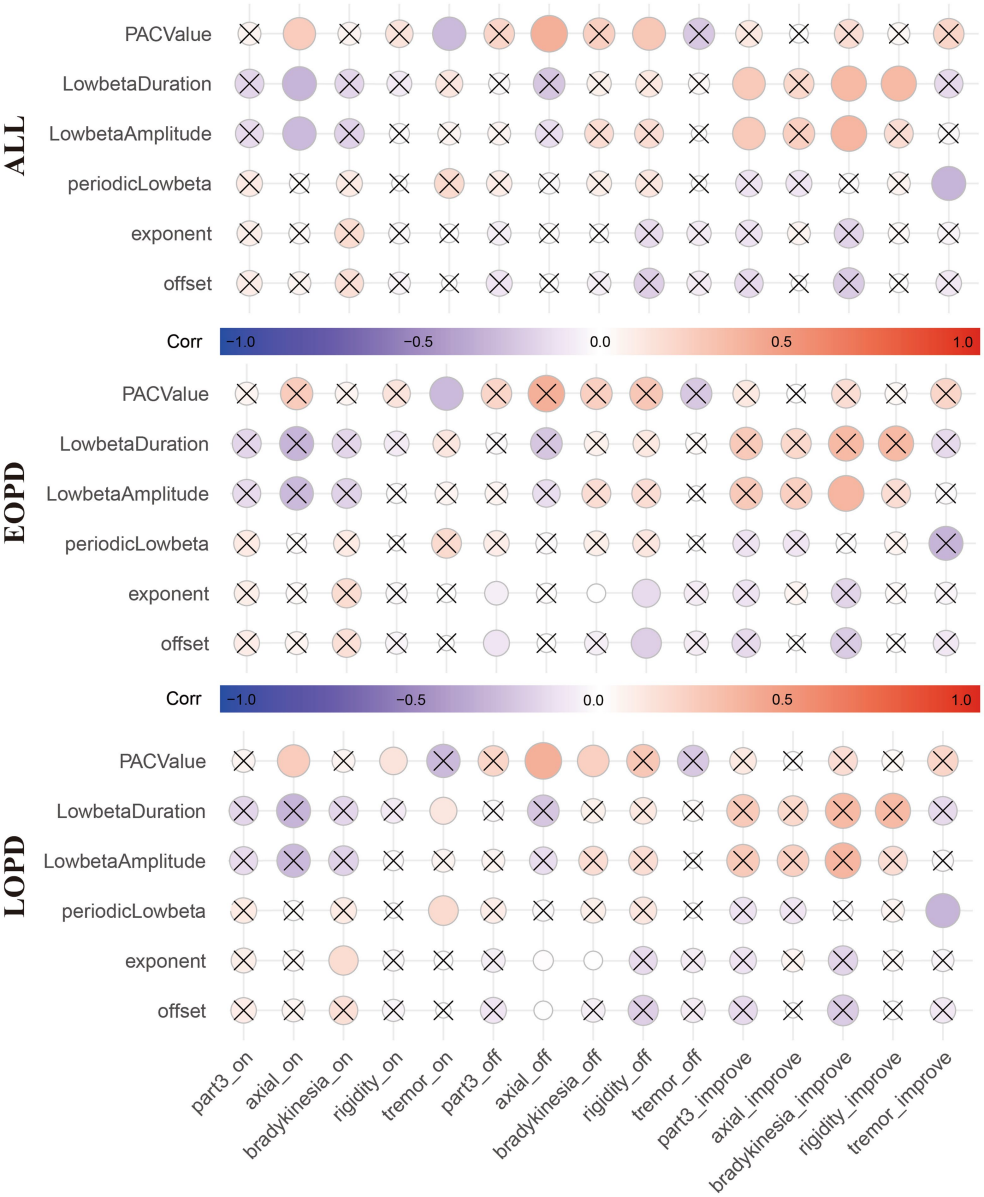
The correlation matrix shows the correlation between the local field potential (LFP) parameter and motor symptom scores (total scores and subdivided scores) and improvement rates in all patients **(Top)**, EOPD **(Middle)** and LOPD **(Bottom)**, respectively. Red represents positive correlation and blue represents negative correlation. Circles with uncorrected *p* values greater than 0.05 were crossed. However, after FDR correction, there were no significant correlations between LFP parameters and the motor scores (including total scores and subdivided scores), neither within whole population nor each group, respectively. *_on indicates Med ON. *_off indicates Med OFF. *_improve indicates improvement rate.

After calculating the results above, we tried to establish their relationship with clinical data. As show in Figure 5, age had significant correlation with offset (*r* = 0.305, FDR corrected *p* = 0.047, Figure 5A) and periodic low-beta PSD (*r* = −0.315, FDR corrected *p* = 0.047, Figure 5C) in whole population. Moreover, exponent (*r* = 0.271, FDR corrected *p* = 0.050, Figure 5B) and PAC value of low beta/fHFO (*r* = −0.284, FDR corrected *p* = 0.050, Figure 5F) were marginal significantly correlated with age. In Figure 6, circles with uncorrected *p* value greater than 0.05 were crossed. However, after FDR correction, there were no significant correlations between LFP parameters and the motor scores (including total scores and subdivided scores), neither within whole population nor each groups, respectively.

## 4. Discussion

To date, there has been no specific research on LFP in EOPD. Here, we demonstrated for the first time several characteristics of the electrophysiological signals recorded from the STN of patients with EOPD.

In the past few decades, the vast majority of electrophysiological investigations have examined the combined periodic oscillatory component and the aperiodic component rather than separating them. Because apparent changes in narrowband power may represent a variety of physiological processes, using narrowband filtering (for example, 13–30 Hz for the beta band) without parameterization can lead to a misrepresentation and incorrect interpretation of physiological phenomena (Donoghue et al., 2020). The aperiodic offset parameter represents total up/down shift of the whole spectrum, and it was proved to be correlated with both the blood-oxygen-level-dependent (BOLD) signal from functional MR imaging (Winawer et al., 2013) and neuronal spiking (Manning et al., 2009). The aperiodic exponent has been related to the integration of the synaptic currents (Buzsáki et al., 2012). Recent studies have found the 1/f-like aperiodic components, which had been considered as noise before, have a unique function and are associated with brain activity (Adelhöfer et al., 2021; Zhang et al., 2021) and age (Voytek et al., 2015; Schaworonkow and Voytek, 2021). Here, our study found the results analogous to previous research, in which offset (*r* = 0.305, corrected *p* = 0.047, Figure 5A) and exponent (*r* = 0.271, corrected *p* = 0.050, Figure 5B) were positively correlated with age.

By investigating the relationship between resting-state EEG activity and the efficiency of cognitive functioning, Ouyang et al. (2020) found that 1/*f* brain activity plays an essential role in cognitive function, and pointed out the necessity of isolating the 1/*f* component from oscillatory activities. Synaptic excitation (E) and inhibition (I), typically represented by quick glutamate and slower GABA inputs, are balanced in neural circuits (Xue et al., 2014). The balance of E:I interaction is essential for the formation of neural oscillations (Atallah and Scanziani, 2009). One study found that reduced E/I ratio resulted in steeper power spectra, reflecting conscious state over time (Gao et al., 2017). In other words, exponent will be lower when E/I ratio increases, and larger when E/I ratio decreases. As in our study, the positive correlation between the aperiodic parameters and age provided evidence that neurons located in the STN of patients with EOPD showed higher excitability than in LOPD. It is noted that symptoms of most PD patients with older onset age result from neurodegeneration, whereas some patients with younger onset age suffer from motor impairment owing to selective dysregulation of dopaminergic production and transfer caused by variable factors such as genetic mutation (Angeli et al., 2013; Pal et al., 2016; Mehanna and Jankovic, 2019; Leuzzi et al., 2021).

The beta pathological oscillatory band has become the most studied band in PD, as it is strongly correlated with movement impairment and can be suppressed by medication (Brown et al., 2001) and DBS (Neumann et al., 2016b). Evidence shows that low-beta activity (13–20 Hz) is more sensitive to levodopa or DBS than high beta (Litvak et al., 2011), and a sub-band (10–14 Hz) partially within the low-beta range is most robustly correlated with UPDRS III total score (Neumann et al., 2016a). Weinberger et al. found positive correlation between the incidence of beta oscillatory neurons and the patient’s response to dopaminergic medications, but not with baseline motor deficits off medication (Weinberger et al., 2006). Similarly in our study, EOPD patients, with higher periodic low-beta power, had better response to anti-parkinsonism medication when compared to LOPD patients, under similar clinical baseline measurements such as LEDD and disease severity (UPDRS III total scores under Med OFF condition) for the PSM we used.

The amount of low-beta burst activity in STN correlates with the progressive decline in movement velocity in a spectrally specific manner, which can better explain motor impairment when compared to average beta power (Lofredi et al., 2019b). The presence, amplitude, and duration of beta bursts in the STN of PD patients were modulated by context and may be crucial for the transformation of physiological information (Torrecillos et al., 2018; Kehnemouyi et al., 2021). The presence of abnormal beta bursts was significantly correlated with the severity of motor impairment in PD, and the distribution of beta burst duration could be changed from long to short by medication and DBS, which represented a more physiological state (Tinkhauser et al., 2017b). They also proved that beta bursts of longer duration were positively correlated and bursts of shorter duration were negatively correlated with motor impairment (Tinkhauser et al., 2017a). Furthermore, Lofredi et al. discovered that frequency-specific low-beta (13–20 Hz) band power and burst duration exhibited substantial relationships with the severity of motor impairments, and that dopamine-related symptom relief occurred simultaneously with reduction of both band power and burst duration (Lofredi et al., 2023). These features indicated low-beta burst duration as a better potential biomarker for aDBS. Our study showed that the average low-beta burst duration of EOPD was significantly longer than that of LOPD. Additionally, EOPD had more longer durations whereas LOPD had more shorter durations. These findings showed that neurons in the STN of patients with EOPD showed more over-synchronization, which was thought to be responsible for pathological beta bursts. Because synchronized neurons are prone to firing simultaneously, they are less likely to fire separately, which means the chances for them to transfer different information individually decrease, leading to restriction of the overall information coding capacity of the circuit (Brittain and Brown, 2014). Our study about burst provided electrophysiological evidence that neurons in the STN of patients with EOPD had higher excitability as we mentioned above. This may be because EOPD involves dysregulation of dopaminergic production and transfer rather than neurodegeneration.

Another potential biomarker for adaptive stimulation is PAC, modulation of the amplitude of high frequency oscillations by the phase of low frequency oscillations (Hwang et al., 2020). By coordinating the activity time of neurons in connected networks, PAC plays an important role in the mechanism for communication within and between neurons in different brain regions (Canolty and Knight, 2010). PAC from different brain regions in PD has been found to be associated with motor impairment (Yin et al., 2022) and cognitive decline (Sacks et al., 2021), and can be affected by medication (Ozturk et al., 2020) and DBS (de Hemptinne et al., 2015; Steiner et al., 2017). Evidence has shown that exaggerated STN PAC between beta band and HFO is correlated with severity of motor impairments (bradykinesia/rigidity), and sub-band low beta is more closely linked to pathology in PD (Lopez-Azcarate et al., 2010; Connolly et al., 2015; van Wijk et al., 2016). Beta/HFO PAC was also found predictive of response to DBS therapy as the PAC with the greatest strength was found to be located in the dorsal STN, where stimulation was most clinically effective (Yang et al., 2014). In our study, with EOPD patients having stronger beta/HFO PAC in the STN, suggesting that neurons in the STN of younger patients may maintain their capacity for information processing and communications.

In our study, the age limit (50 years) was defined based on past experience, habits, epidemiology and clinical characteristics. There were still doubts that this age limit may not truly separate the groups so that the results should be interpreted cautiously. Continuous recordings from DBS device with sensing technology (i.e., Percept™ PC Neurostimulator, Medtronic) may provide a chance to get insight of the electrophysiological changes as age grows. More researches are needed to deepen our understanding of the relationship between age and disease.

## 5. Limitations

There were several limitations in our study. First, the data were recorded under the resting state, and the electrophysiological properties of the STN may vary in different movement states, which was not fully evaluated. Second, postoperative data were not complete and the follow-up period was short, so we were unable to identify the relationship between symptom improvement and electrophysiological signals of the STN. Third, we lacked patient genetic information, so we were unable to explore the genetic mechanism underlying the electrophysiological phenotype, which is worth exploring in the future.

In addition, the fact that the different median ages of the groups differed raised another important concern that some of the observed differences may be caused by age differences, when the patients underwent DBS and the signals were recorded, rather than disease factors. Further research of recordings from patients with other diseases or EOPD patients after aging may help ease this issue.

## 6. Conclusion

Our study revealed the electrophysiological features of the STN in EOPD for the first time. It is widely acknowledged that aDBS will play an inevitable role in the management of functional disease of the central nervous system, and sensitive and specific electrophysiological biomarkers in cohorts of different ages are required. We found that low-beta activity in the STN of patients with EOPD had different characteristics to LOPD, which may be because the different pathological processes in EOPD cause neurons to exhibit higher excitability. This should be considered when applying aDBS on patients of different ages.

## Data availability statement

The original contributions presented in the study are included in the article/Supplementary material, further inquiries can be directed to the corresponding authors.

## Ethics statement

The studies involving human participants were reviewed and approved by institutional review board of Beijing Tiantan Hospital. The patients/participants provided their written informed consent to participate in this study.

## Author contributions

JZ performed conceptual design of experiments and experiment’s integration, co-wrote the manuscript, and performed all the DBS surgery. AY checked the MER and postoperative CT scan. DW designed, performed, and analyzed electrophysiology recordings, and co-wrote the manuscript. BZ wrote the custumed code in MATLAB and R studio. HX co-wrote the manuscript. YX plotted the first three figures in MATLAB. ZY wrote the code in Python to calculate the PAC value. YB performed the PSM in SPSS. HF, QZ, and DL re-checked the data and analyzed results. TH, XZ, YJ, and QA did the clinical assessments and data collection. All authors contributed to the article and approved the submitted version.

## Funding

This work was funded by the National Natural Science Foundation of China (81830033 and 81870888).

## Conflict of interest

The authors declare that the research was conducted in the absence of any commercial or financial relationships that could be construed as a potential conflict of interest.

## Publisher’s note

All claims expressed in this article are solely those of the authors and do not necessarily represent those of their affiliated organizations, or those of the publisher, the editors and the reviewers. Any product that may be evaluated in this article, or claim that may be made by its manufacturer, is not guaranteed or endorsed by the publisher.

## Footnotes

^1^ https://www.fieldtriptoolbox.org/

^2^ https://etiennecmb.github.io/tensorpac/
